# Retrospective Observation of Low-Dose Rituximab Treatment in Chinese Patients With Neuromyelitis Optica Spectrum Disorders in a Real-World Setting

**DOI:** 10.3389/fneur.2020.00642

**Published:** 2020-07-07

**Authors:** Haibing Xiao, Wenshuang Zeng, Ling Li, Lina Li, Yuzhen Cui, Jie Wang, Jinhao Ye, Qingyan Yang

**Affiliations:** Neurology, Department of Medicine, The University of Hong Kong-Shenzhen Hospital, Shenzhen, China

**Keywords:** neuromyelitis optica spectrum disorders, rituximab, aquaporin 4 antibody, annualized relapse rate, multiple sclerosis

## Abstract

**Objective:** This study aimed to investigate the efficacy and safety of low-dose rituximab (RTX) in the treatment of neuromyelitis optica spectrum disorders (NMOSD) patients.

**Methods:** NMOSD patients were treated with RTX at ~25% of the standard dose. The annualized relapse rate (ARR), expanded disability status scale (EDSS) score, visual function system scale (VFSS) and length of spinal cord lesions before and after treatment were statistically compared. The dynamic changes in the proportion of CD19^+^ B lymphocytes after treatment were monitored, and adverse reactions were recorded.

**Results:** In total, 36 NMOSD patients who received a low-dose RTX treatment (375-mg/m^2^ induction dose and 500 mg every 6 months) were recruited. The mean follow-up time after the RTX treatment was 19.83 ± 7.74 months. After the treatment, the ARR decreased from 1.97 ± 1.93 to 0.12 ± 0.32, the EDSS score decreased from 3.43 ± 1.49 to 3.10 ± 1.88, and the spinal cord lesion length decreased from 5.54 ± 3.96 to 4.31 ± 3.73. These differences were all statistically significant. The subgroup analysis of the patients who had previously received non-steroidal immunosuppressants (NSISs) (*n* = 20) showed that after the RTX treatment, the ARR decreased from 0.66 ± 0.51 to 0.08 ± 0.26, the EDSS score decreased from 3.65 ± 1.22 to 3.40 ± 1.99, and the spinal cord lesion length decreased from 5.68 ± 3.73 to 4.21 ± 3.58. These differences were all statistically significant. The VFSS scores did not show a significant change. The Kaplan-Meier analysis showed that low-dose RTX significantly delayed recurrence, which was also observed in the subgroup analysis of patients who previously received NSISs. Five relapses in 5 cases were noted after the low-dose RTX administration, and the percentage of CD19^+^ B cells remained < 1% in 3 cases during relapse. During the RTX treatment and subsequent follow-up, 8 (22.2%) patients reported adverse reactions, all of which were minor.

**Conclusion:** Low-dose RTX is an effective and safe treatment method for NMOSDs. This method is worth popularizing in developing countries or regions, especially in areas where RTX is not covered by medical insurance.

## Introduction

Inflammatory demyelinating diseases of the central nervous system are the leading causes of neurological disability in young people. Owing to the low prevalence of multiple sclerosis (MS) in Asian populations (1), neuromyelitis optica spectrum disorders (NMOSDs) have become the most prominent inflammatory demyelinating diseases of the central nervous system in the East Asian population (2). Discovery of the disease-specific aquaporin 4 (AQP4) antibody (3, 4) has accelerated our understanding of the disease, and the name of the disease has also been extended from neuromyelitis optica (NMO) to NMOSD (5, 6).

The characteristic clinical manifestations of NMOSDs include attacks of optic neuritis, longitudinally extensive transverse myelitis or brainstem encephalitis (especially area postrema syndrome), and diencephalon and brain involvement is observed in some patients (6, 7). The average annualized relapse rate (ARR) of NMOSDs has reached 2.2 (8), and the relapse rate is higher than that of MS. After repeated episodes, patients are often left with significant neurological disability.

The main treatment regimen for NMOSDs in the acute stage is high-dose intravenous methylprednisolone or plasmapheresis and intravenous immunoglobulin (IVIG), if necessary. The main purpose of treatment during remission is to prevent relapses, thus delaying the progression of neurological disability. The commonly used first-line drugs include rituximab (RTX), azathioprine, and mycophenolate mofetil; the second-line drugs include cyclophosphamide, methotrexate and tacrolimus (9, 10).

Most NMOSD patients are females; thus, aesthetic and pregnancy needs will affect the choice of medication. Disadvantages such as potential liver toxicity and bone marrow toxicity (11), frequent routine blood and liver function monitoring, and insufficient efficacy (8) have limited the use of azathioprine. In developed countries, as a first-line recommendation, RTX has been widely used to prevent the relapse of NMOSDs (9), not only because RTX has better efficacy and safety but also because of the medical insurance support in these countries. In Europe and the United States, neurologists directly prescribe the dose of RTX to treat lymphoma (375 mg/m^2^ weekly for 4 weeks or 1 g every 2 weeks twice). RTX application in the treatment of NMOSDs in China is not covered by medical insurance. Therefore, a high RTX dose has a high cost for patients. Whether NMOSD patients need such high RTX doses is also a question that concerns many physicians.

For other autoimmune diseases, such as myasthenia gravis (MG) and autoimmune pancytopenia, precedents have been established for successful treatment with low-dose RTX (12, 13). Considering that autoimmune diseases are not tumors, the requirement for B lymphocyte clearance is not as high as in the treatment of lymphoma. We also believe that the treatment of NMOSDs with low-dose RTX is logically reasonable.

A reduced dose (~20–25% of the standard dose) of RTX reportedly has therapeutic value for NMOSDs (14–16). However, the number of patients included in such studies was generally low. Therefore, the efficacy and safety of reduced-dose therapy still require more research and observation.

This paper reviewed a series of NMOSD patients who received RTX treatment in our hospital between January 2016 and March 2020. Our analysis suggests that in real-world clinical practice, low-dose RTX treatment for Chinese NMOSD patients has relatively good efficacy and safety.

## Method

### Patients, Treatment, and Evaluations

A retrospective analysis was performed on NMOSD patients who received RTX treatment at the Neurology Department, Department of Medicine, the University of Hong Kong-Shenzhen Hospital (HKU-SZH), between January 2016 and March 2020. The diagnostic criteria for NMOSDs refer to the international diagnostic standards established in 2015 (6). MS patients and myelin oligodendrocyte glycoprotein encephalomyelitis patients were excluded from this study. Demographic, clinical, imaging, and laboratory indicators were collected. The number of episodes of the patients was recorded to calculate the ARR. In the overall analysis, the ARR for individual cases in prespecified periods was calculated as the number of relapses divided by the number of months before and after RTX initiation and multiplied by 12. The expanded disability status scale (EDSS) was used to assess the overall neurological function of patients. The visual function system scale (VFSS) of EDSS was used to evaluate the functional status of each patient's visual system.

All patients received 500–1,000 mg methylprednisolone intravenous therapy for 5 days in the acute phase. If the effect was not satisfactory after 2 weeks, plasmapheresis/IVIG therapy was recommended. During remission, some patients converted from azathioprine, prednisone, mycophenolate mofetil, or tacrolimus to RTX for various reasons, whereas some patients directly chose RTX as an initial preventive drug. The induction dose of RTX was 375 mg/m^2^ per single use. In our series, the doses were 500–700 mg (to be cautious, we started with a dose of 100 mg, and if no severe side effects developed, the remaining dose was given within 24 h). Then, a 500-mg injection was repeated once every 6 months. We developed this low-dose regimen because we hope to allow more patients to be able to afford RTX treatment as RTX is expensive in China and not covered by the national health insurance for NMOSDs. Before each RTX injection, we preadministered acetaminophen 1,000 mg orally, hydrocortisone 100 mg intravenously, and chlorpheniramine 10 mg intravenously to reduce RTX infusion-related reactions.

After RTX treatment, the patients were followed up every 2-3 months. The disease episodes were recorded, and the EDSS score, VFSS score, and the proportion of CD19^+^ B lymphocytes were measured. The patients underwent a magnetic resonance imaging (MRI) scan of the brain and spinal cord every 6 months if no recurrence was identified. In cases of recurrence, MRI was performed within 1 week after relapse.

This is a retrospective analysis of real-world clinical practice; therefore, we did not set up a control group. To compensate for this shortcoming, we performed a subgroup analysis of some patients who had previously used non-steroidal immunosuppressants (NSISs) along with an overall retrospective analysis. In this subgroup analysis, the prespecified periods for calculating the ARR were the durations of NSIS use and RTX treatment. This subgroup analysis helped us compare the efficacy between RTX and other NSISs.

### Statistical Analysis

We used Excel and SPSS 17.0 for statistical analysis. The data normality was assessed by the Kolmogorov–Smirnov test, and the mean ± standard deviation (SD) is used to describe the continuous data with a normal distribution. A paired *T*-test was used to compare the ARR, disability scores, and spinal cord lesions before and after RTX treatment. An independent *T*-test was used to compare demographic characteristics between subgroups. The Kaplan–Meier survival curve was used to analyse the time to the first relapse for different treatment regimens (or treatment stages), and the log-rank test was used to compare differences between different regimens (or different stages).

## Results

### Basic Information

A total of 36 NMOSD patients were included, all of whom were Han Chinese, including 3 males and 33 females (male: female ratio of 1:11). The mean onset age was 36.6 ± 14.3 years. A total of 32 patients were AQP4 antibody positive, accounting for 90% of the cases. Among these patients, 8 (22.2%) had optic neuritis, 11 (30.6%) had myelitis, 6 had area postrema syndrome (16.7%), and 11 had other syndromes, including combinations of the aforementioned syndromes, other brainstem syndromes, and diencephalic or cerebral syndromes (accounting for 30.6% of the patients). The mean disease course was 61.72 ± 47.90 months. Eight patients (22.2%) had other systemic immune diseases, including 5 cases of Sjogren's syndrome, 1 case of Hashimoto's thyroiditis, 1 case of mixed connective tissue disease, and 1 case of rheumatoid arthritis (Tables 1, 2).

**Table 1:** Characteristics of the case series with neuromyelitis optic spectrum disorders (*n* = 36).

Female vs. male: 33 vs. 3
Aquaporin 4 antibody positive: 32 (90%)
Onset syndrome
Optic neuritis 8 (22.2%)
Myelitis 11 (30.6%)
Area postrema syndrome 6 (16.7%)
Others 11 (30.6%, combination of above, brainstem, diencephalic, and/or cerebral syndrome)
Autoimmune comorbidities 8 (22.2%)
Sjogren syndrome 5
Others 3 (1 rheumatoid arthritis, 1 mixed connective tissue disease, 1 Hashimoto's thyroiditis)
Non-steroidal immunosuppressants before RTX: 20 (55.6%)
Azathioprine 9
Mycophenolate mofetil 7
Cyclophosphamide 3
Tacrolimus 1

**Table 2:** Demographic characteristics in the overall case series and subgroups.

	**Onset age (years)**	**Total disease duration (months)**	**Disease duration before RTX initiation (months)**	**Length of NSIS therapy (months)**	**ARR before RTX initiation**	**ARR during NSIS therapy**	**Follow-up after RTX initiation (months)**
Overall	36.6 ± 14.31	61.72 ± 47.90	42.14 ± 47.55	N.A.	1.97 ± 1.93	N.A.	19.83 ± 7.74
NSIS subgroup	34.05 ± 14.99	75.75 ± 53.19	55.35 ± 51.93	31.75 ± 18.20	1.20 ± 0.78	0.66 ± 0.51	20.85 ± 8.12
Non-NSIS subgroup	39.88 ± 12.70	44.19 ± 32.74	25.63 ± 35.01^*^	N.A.	2.92 ± 2.40	N.A.	18.56 ± 7.03

* *P < 0.05 compared with the NSIS subgroup*.

*NSISs, nonsteroidal immunosuppressants; ARR, annualized relapse rate; RTX, rituximab*.

Twenty patients received NSISs (55.6%) before receiving RTX, including azathioprine (9 patients), mycophenolate mofetil (7 patients), cyclophosphamide (3 patients), and tacrolimus (1 patient). Among the patients who switched from other therapies to RTX, 9 patients switched due to unsatisfactory efficacy, 8 patients switched due to intolerable side effects, and 3 patients switched for convenience or because of a stronger belief in the efficacy of RTX. After the RTX treatment, the mean follow-up period was 19.83 ± 7.74 months (Tables 1, 2).

The demographic characteristics and relevant clinical data in the overall case series, the NSIS subgroup, and the non-NSIS subgroup are shown in Table 2. The disease duration before RTX initiation in the non-NSIS subgroup was significantly shorter than that in the NSIS subgroup.

### Changes in the ARR, EDSS, and VFSS Scores in the Overall Patient Series Before and After Treatment

The overall analysis of the case series showed that the ARR of the patients before low-dose RTX treatment was 1.97 ± 1.93, and the ARR after treatment was 0.12 ± 0.32, reflecting a significant improvement. The EDSS score was 3.43 ± 1.49 before the RTX treatment and 3.10 ± 1.88 at the final follow-up after the RTX treatment, also reflecting a significant improvement (Table 3). The VFSS score of the patients was 1.50 ± 1.76 before the low-dose RTX treatment and 1.43 ± 1.73 at the final follow-up after the RTX treatment, revealing no significant difference.

**Table 3:** Changes in the ARR, EDSS, VFSS, and length of spinal cord lesions before and after treatment with low-dose RTX in the overall sample.

***n* = 36**	**ARR**	**EDSS**	**VFSS**	**Length of spinal cord lesions**
Before RTX treatment	1.97 ± 1.93	3.43 ± 1.49	1.50 ± 1.76	5.54 ± 3.96
After RTX treatment	0.12 ± 0.32	3.10 ± 1.88	1.43 ± 1.73	4.31 ± 3.73
Significance level	0.000	0.013	0.169	0.001^*^

* *Data from 35 cases were analyzed, 1 patient died, and no recent MRI was available*.

*ARR, annualized relapse rate; EDSS, expanded disability status scale; VFSS, visual function system scale; RTX, rituximab*.

We also compared the length of spinal cord lesions, as observed on MRI, before and after RTX treatment. Before the low-dose RTX application, the mean length of the spinal cord lesions was 5.54 ± 3.96 vertebral segments, and the length at the final follow-up after treatment was 4.31 ± 3.73, reflecting a significant difference (Table 3).

### Subgroup Analysis

In this case series, 20 patients had received other NSISs before receiving low-dose RTX to prevent recurrence. This subgroup of patients switched to low-dose RTX treatment for various reasons. The analysis showed that before RTX treatment, the ARR of the patients in this subgroup was 0.66 ± 0.51, and the ARR was 0.08 ± 0.26 after treatment, reflecting a significant difference (Table 4).

**Table 4:** Subgroup analysis comparing the efficacy of low-dose RTX and other NSISs.

***n* = 20**	**ARR**	**EDSS**	**VFSS**	**Length of spinal cord lesion**
NSISs	0.66 ± 0.51	3.65 ± 1.22	2.10 ± 2.02	5.68 ± 3.73
Low-dose RTX	0.08 ± 0.26	3.40 ± 1.99	2.05 ± 2.01	4.21 ± 3.58
Significance	0.000	0.039	0.33	0.006^*^

* *Data from 19 cases were analyzed, 1 patient died, and no recent MRI was available*.

*NSISs, non-steroidal immunosuppressants; ARR, annualized relapse rate; EDSS, expanded disability status scale; VFSS, visual function system scale; RTX, rituximab*.

The EDSS scores of this subgroup were also analyzed. The results showed that before RTX treatment, the patients had an EDSS score of 3.65 ± 1.22, and at the final follow-up after RTX treatment, the EDSS score was 3.40 ± 1.99, representing a statistically significant difference (Table 4). The VFSS score of this subgroup before low-dose RTX treatment was 2.10 ± 2.02, and at the last follow-up after treatment it was 2.05 ± 2.01, demonstrating no significant difference.

The analysis of the length of the spinal cord lesions revealed by MRI in this subgroup of patients showed that the mean lengths of the spinal cord lesions were 5.68 ± 3.73 before the low-dose RTX application and 4.21 ± 3.58 at the final follow-up after the RTX injection, revealing a significant difference (Table 4).

### Effect of Low-Dose RTX on the Relapse Time of Patients

**Figure 1:**
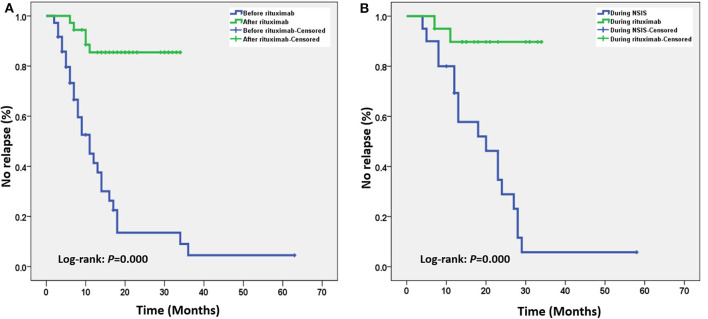
**(A)** Kaplan–Meier survival curve analysis of the overall case series. After low-dose rituximab (RTX) treatment, the time to the first relapse was significantly delayed. **(B)** Subgroup analysis of patients who received other non-steroidal immunosuppressants (NSISs) before receiving RTX showed that after low-dose RTX treatment, the time to the first relapse was still significantly delayed.

Kaplan-Meier survival analysis showed that, in the overall case series (*n* = 36), low-dose RTX treatment significantly delayed the time to first relapse (Figure 1A). Patients who had used other NSISs before RTX treatment were extracted for subgroup analysis (*n* = 20), and low-dose RTX treatment still delayed the time to first relapse compared to that of the NSIS treatment phase (Figure 1B).

### Dynamic Changes in CD19^+^ B Lymphocytes

After RTX treatment, lymphocyte subsets were analyzed at 1 month, 3 months, and 6 months after injection. Because of the limitations of hospital testing conditions, we mainly detected the proportion of CD19^+^ B lymphocytes. After the first RTX injection, all patients (100%) had a B lymphocyte proportion below 1% in the first month, and 25 (69.4%) had 0% B lymphocytes. At the 3rd month, the B lymphocytes of 35 patients (97.2%) were still below 1%, and 17 (47.2%) still had 0% B lymphocytes; at the 6th month, 14 (38.9%) had B lymphocytes below 1%, and 3 (8.3%) had 0% B lymphocytes (Table 5).

**Table 5:** Dynamic changes in B cell depletion after low-dose rituximab (RTX) injection.

**Proportion of CD19^**+**^ B cells**	**Number of cases in which B cells reached different degrees of depletion at different time points (%)**		
	**1 month after RTX injection**	**3 months after RTX injection**	**6 months after RTX injection**
< 1%	36 (100%)	35 (97.2%)	14 (38.9%)
0%	25 (69.4%)	17 (47.2%)	3 (8.3%)

In our case series, a total of 5 patients each had 1 relapse after RTX injection. One patient relapsed in the 6th month after the first round of RTX injection after the B lymphocyte ratio reached > 1%. One patient relapsed within 1 month after the 2nd round of RTX injection, and 2 other patients relapsed 4 months after the 2nd round of RTX injection. These 3 patients had a B lymphocyte ratio < 1% upon relapse. One patient returned to her home village in Hubei province for the Spring Festival and experienced a serious relapse of myelitis and brainstem encephalitis in the 5th month after the 2nd round of RTX injection. Because of the Coronavirus disease 2019 (COVID-19) epidemic and the city lockdown, she could not receive support from a professional team or a ventilator, and she died on 6 March 2020. No lymphocyte analysis or MRI was performed at the time of her relapse.

### Safety

A total of 8 patients (22.2%) reported adverse events. Five had injection-related adverse reactions (3 with rash/pruritus, 2 with a low fever), and 3 reported urinary tract infection within 2 weeks after injection. The above symptoms subsided after symptomatic or supportive treatment.

## Discussion

Commonly used immunosuppressive agents in the prevention and treatment of NMOSDs include azathioprine, prednisone, mycophenolate mofetil, and cyclophosphamide. Due to insufficient efficacy and many side effects, some patients and physicians seek stronger or safer treatment options. RTX depletes B lymphocytes by recognizing CD20 to exert its therapeutic functions. On one hand, RTX removes B lymphocytes and reduces AQP4 antibody production; on the other hand, RTX may also play a long-term immunotherapy role by changing the composition of newly proliferated B lymphocytes and restructuring the ratio of regulatory B (Breg)/memory B (Bmem) lymphocytes (17). Due to its effectiveness and safety, RTX has gradually become the mainstream treatment method in developed countries and regions.

RTX was originally developed as a therapeutic drug for non-Hodgkin's lymphoma. Subsequently, RTX was gradually expanded to other autoimmune diseases, including systemic lupus erythematosus and rheumatoid arthritis. When neurologists use RTX to treat neurological diseases such as NMO, MS, and MG, the treatment dose and frequency of lymphoma are used for reference. A meta-analysis in 2016 (18) included a total of 438 NMOSD patients in 46 studies, and 94.2% of the studies were based on the lymphoma treatment regimen (375 mg/m^2^ weekly for 4 weeks or 1 g every 2 weeks). This treatment was shown to be effective as demonstrated by a decrease in the mean ARR of 0.79 ± 0.15 after the RTX injection, but the incidence of adverse reactions was high. The analysis showed that 26% of patients reported adverse reactions, 10.3% of whom had infusion-related adverse reactions, while 9.1% had infections, 4.6% had persistent leukopenia, 0.5% had posterior reversible encephalopathy, and 1.6% died. A recently reported multicenter, randomized, double-blind, placebo-controlled study including 19 patients showed that a high RTX dose prevented relapses for 72 weeks in patients with NMOSDs who were AQP4 antibody positive (19). However, this study is limited by its small sample size and the inclusion of participants with mild disease activity (EDSS < = 7). Shaygannejad and colleagues reported a prospective study exploring the long-term tolerability, safety and efficacy of RTX for NMOSDs (20). The induction dose was 500 mg/week for 4 consecutive weeks, and the maintenance regimen was 1000 mg every 6 months. Their results showed that during the 1-2-year observation period, the ARR decreased from 0.26 ± 0.54 to 0, the EDSS decreased from 4.1 ± 1.8 to 3.1 ± 1.8, and 31.8% of the patients had a mild infusion reaction.

In developing countries such as China, RTX is expensive and not covered by medical insurance. Patients expect lower RTX doses but equivalent efficacy to reduce their out-of-pocket cost. From the perspective of disease differentiation, to treat lymphoma, a type of cancer, doctors prescribe high RTX doses and pursue complete B lymphocyte depletion. However, to treat NMO, an immune disease, administering relatively low RTX doses is theoretically feasible to significantly reduce the number of B lymphocytes while allowing the presence of few B lymphocytes, thereby reducing the recurrence of autoantibody-induced demyelinating events. Low-dose RTX treatment has been effective in other autoimmune diseases, such as MG (12) and pancytopenia (13).

The most concerning issue for doctors and patients is whether low-dose RTX is sufficiently effective to reduce disease recurrence and disability accumulation, but very few studies on this topic are available. In 2013, Yang et al. reported that 100-mg RTX administration in 3 consecutive injections each week showed sufficient efficacy (14), but their study included only 5 patients. CD19^+^ B lymphocytes were still below 1%, and memory B lymphocytes were < 0.5% in 1 patient 53 weeks after RTX injection. Zhang et al. (21) reported 31 NMOSD patients who received low-dose RTX treatment (100 mg weekly 3 times, repeated 3 times after a 4-week interval). Repeated injections were performed if CD19^+^ cells had increased to > 1%. The results showed that the effectiveness and safety of this treatment were better than those of azathioprine treatment. In 2018, Lin et al. described 14 patients who received low-dose RTX treatment (375 mg/m^2^ once and repeated every 6 months) (16). They found that compared with other immunosuppressants, low-dose RTX significantly reduced the ARR and EDSS and prolonged the recurrence interval after treatment. The drug administration regimen of Lin's study was very similar to ours. Lu et al. recently reported another retrospective study including 20 NMOSD patients treated with low-dose RTX (22). In this study, the median RTX dose was 500 mg, and the median interval was 6.1 months. This study showed that the median ARR decreased by 90% after RTX initiation, and only mild infections and allergies were reported.

The drug administration method used in our study was more convenient than the multiple drug administration schemes of Yang (14) and Zhang (21). Compared with the study by Lin et al., we evaluated a larger patient population receiving RTX (16). Our results showed that low-dose RTX significantly reduced the ARR in both the overall case series and the subgroup that previously received NSIS. In the analysis of the EDSS, we also find a similar reduction, although the statistical significance was lower than that in the ARR analysis. However, this improvement in the EDSS cannot be simply interpreted as a therapeutic effect of RTX. A more plausible explanation is that some patients in our cohort started receiving RTX shortly after relapse. As we know, disability in NMOSDs is almost entirely driven by relapses. Thus, the pre-RTX EDSS did not systematically account for sustained disability; consequently, the EDSS improvement cannot be directly attributed to RTX. The same problem applies to the length of spinal cord lesions. Indeed, it is well-known that in NMOSDs, the length of spinal cord lesions tends to decrease over time after the latest myelitis. In the absence of other treatment comparisons, even if this reduction in the length of spinal cord lesions in our study is significant, this reduction should not be interpreted simply as a direct benefit of RTX. Regarding the VFSS, which is an indicator of visual functional disability, there were no changes before or after the RTX treatment, which may be related to a poor compensatory potential after optic nerve damage. However, regarding the most direct indicator of disease activity, i.e., the ARR, low-dose RTX has a definite and significant effect in our study. In addition, when we performed the Kaplan-Meier analysis, we found that low-dose RTX significantly delayed the time to relapse in the NMOSD patients in both the overall series and the subgroup that previously received NSISs. Notably, the efficacy observed in our case group was similar to that in other studies using standard-dose therapy (18, 19, 23, 24), although the methods used in each study were heterogeneous.

After RTX treatment of NMOSDs, no consensus on determining the timing of repeat injections and monitoring the B lymphocyte subpopulation is available. Some scholars have monitored the proportion of memory B lymphocytes to guide subsequent injections (25). Strategies adopted by some scholars include maintaining CD19^+^ B lymphocytes below 1% or repeating drug injections once every 6 months (16). Novi and colleagues reported an observational-retrospective multicenter study evaluating the efficacy of different RTX therapeutic strategies in patients with NMOSDs (26) who were treated with different high-dose RTX regimens in 21 Italian centers and 1 Swiss center. The results showed that the ARR decreased from 1.7 to 0.19 after RTX initiation. Regarding different induction and maintenance regimens, IND-B (375-mg/m^2^/week infusions for 1 month) and M-A (fixed time-point infusions of 1000 mg every 6 months) may be less effective strategies as both the ARR and the time to the first relapse analyses showed a trend toward increased disease activity with IND-B and M-A. No patients receiving M-B2 (375-mg/m^2^ reinfusions based on CD27^+^ CD19^+^ memory B cell re-emergence when this population exceeded 0.05%) experienced relapses during the follow-up. Our laboratory was unable to detect CD27 to determine the proportion of memory B lymphocytes; therefore, we only monitored the proportion of CD19^+^ B cells and empirically suggested that patients should repeat the injection every 6 months.

In practice, we found that after RTX injection, the percentage of the patients with B lymphocytes that dropped below 1% in the first month was 100%, 25 of whom had 0% B lymphocytes. Subsequently, the percentage of B lymphocytes in patients gradually recovered. At the 6th month, B lymphocytes remained below 1% in 14 patients (38.9%). In our case group, a total of 5 patients each had 1 recurrence after RTX injection. One patient relapsed at the 6th month after injection when B lymphocytes were already > 1%. Three patients relapsed within 6 months after injection, and the percentage of lymphocytes during relapse was < 1%. One patient relapsed and eventually died because she could not receive support from a ventilator or a professional team due to COVID-19 and the related lockdown measures. Lymphocyte subset analysis was not performed when she relapsed. These results suggest that close monitoring and limiting peripheral blood B lymphocytes to < 1% in NMOSD patients receiving RTX treatment may not reduce the risk of recurrence; monitoring and controlling CD19^+^ CD27^+^ memory B cells to <0.05% may be a better choice (26). We also hypothesize that relapse is partly related to some long-lived plasma cells in tissues. These plasma cells did not express CD20 and therefore could not be cleared by RTX. Future novel drugs targeting CD19^+^ cells, if possible, may have better prospects.

Our study found that only 8 patients (22.2%) reported adverse reactions at the time of or at the late stage of RTX injection, and all the adverse reactions were very mild. This rate is significantly lower than the incidence of adverse reactions to standard treatment (18) and may be related to our use of a relatively low RTX dose and 3 kinds of drugs to prevent side effects in advance. This finding also suggests that RTX is generally a safe drug. Ocrelizumab, a newly introduced full human-derived protein structured CD20^+^ B lymphocyte scavenger, may further reduce the side effects of the treatment. However, among the 36 patients with an average follow-up of 19.8 months under RTX, no respiratory tract infection (or other type of infection, except for urinary infection in 3 cases) is reported. Even if the dose of RTX is low, this finding is slightly surprising. Our study is retrospective, and the data were collected from patient records archived in our hospital. We cannot rule out the possibility that some patients had some self-limiting infections, such as upper respiratory tract infections, that were not reported to the doctors or recorded in the medical files, resulting in a slight underestimation of infection events.

NMOSD patients have been reported to show obvious hypogammaglobulinemia after long-term high-dose RTX treatment, which undoubtedly increases the risk of complications such as infection (27). Because our cohort received a lower RTX dose, this risk may be reduced. However, our observation period was not sufficiently long, and we did not monitor blood gamma globulin in every patient in advance; thus, we cannot draw any conclusions on this topic. However, we believe that regardless of whether a low dose or a high dose is used, long-term gamma globulin monitoring is necessary for all NMOSD patients receiving RTX treatment in future clinical practice.

Our study has some limitations: (1) Our sample size was not large, and the follow-up was not long; (2) The disease course before receiving RTX was short in some cases, and the calculated ARR may be overestimated, potentially leading to overestimation of RTX efficacy; (3) Because of the medical cost, most of our patients selected low-dose RTX treatment, and we did not have a high-dose group as a control. These limitations may render our conclusions less convincing. We call for studies with larger sample sizes, longer follow-ups, and high-dose control groups to further confirm the efficacy and safety of low-dose RTX for the treatment of NMOSDs.

In summary, we retrospectively reported the protocol and results of low-dose RTX treatment in 36 NMOSD patients in a real-world setting. Our findings suggest that low RTX doses can significantly reduce the ARR of NMOSD patients, which may improve overall neurological function to some extent and stabilize the visual function of patients. This benefit was also observed in the subgroup of patients who had previously received other NSIS agents. In the treatment of NMOSDs with low-dose RTX, close monitoring and control of the B lymphocyte ratio to <1% may have no definite effect on reducing the relapse rate in our cohort. According to the literature, monitoring and controlling CD19^+^ CD27^+^ memory B cells to <0.05% may be a better choice. Low-dose RTX treatment for NMOSDs is safe and worthy of promotion in developing countries or regions, especially in areas where RTX is still not covered by medical insurance.

## Data Availability Statement

The datasets generated for this study are available on request to the corresponding author.

## Ethics Statement

The studies involving human participants were reviewed and approved by Ethics committee of the University Hong Kong-Shenzhen Hospital. The patients/participants provided their written informed consent to participate in this study.

## Author Contributions

HX was responsible for the patient follow-up, study design, data analysis, and completion of the manuscript. WZ, LingL, LinaL, YC, JW, JY, and QY were responsible for treatment and data collection. All authors contributed to the article and approved the submitted version.

## Conflict of Interest

The authors declare that the research was conducted in the absence of any commercial or financial relationships that could be construed as a potential conflict of interest.
